# SOX11 promotes invasive growth and ductal carcinoma in situ progression

**DOI:** 10.1002/path.4939

**Published:** 2017-08-22

**Authors:** Erik Oliemuller, Naoko Kogata, Philip Bland, Divya Kriplani, Frances Daley, Syed Haider, Vandna Shah, Elinor J Sawyer, Beatrice A Howard

**Affiliations:** ^1^ The Breast Cancer Now Toby Robins Research Centre, Division of Breast Cancer Research The Institute of Cancer Research London UK; ^2^ Research Oncology, Guy's Hospital King's College London London UK

**Keywords:** SOX11, DCIS, embryonic mammary marker, mammary progenitor/stem cells, invasion, ALDH1A1

## Abstract

Here, we show that SOX11, an embryonic mammary marker that is normally silent in postnatal breast cells, is expressed in many oestrogen receptor‐negative preinvasive ductal carcinoma in situ (DCIS) lesions. Mature mammary epithelial cells engineered to express SOX11 showed alterations in progenitor cell populations, including an expanded basal‐like population with increased aldehyde dehydrogenase (ALDH) activity, and increased mammosphere‐forming capacity. DCIS.com cells engineered to express SOX11 showed increased ALDH activity, which is a feature of cancer stem cells. The CD44+/CD24–/ALDH+ cell population was increased in DCIS.com cells that expressed SOX11. Upregulating SOX11 expression in DCIS.com cells led to increased invasive growth both in vitro and when they were injected intraductally in a mouse model of DCIS that recapitulates human disease. Invasive lesions formed sooner and tumour growth was augmented in vivo, suggesting that SOX11 contributes to the progression of DCIS to invasive breast cancer. We identified potential downstream effectors of SOX11 during both microinvasive and invasive tumour growth stages, including several with established links to regulation of progenitor cell function and prenatal developmental growth. Our findings suggest that SOX11 is a potential biomarker for DCIS lesions containing cells harbouring distinct biological features that are likely to progress to invasive breast cancer. © 2017 The Authors. The Journal of Pathology published by John Wiley & Sons Ltd on behalf of Pathological Society of Great Britain and Ireland.

## Introduction

Embryonic breast epithelial cells constitute a unique cell population composed of undifferentiated and highly plastic progenitor cells that ultimately give rise to all other postnatal breast epithelial cells 1. There is increasing evidence that cancer stem or stem‐like cells exist and perpetuate the growth of cancer cells after therapy in many solid tumours 2, 3. Cancer stem cells identified in the skin, gut and brain are very similar to the healthy stem cells responsible for growing and renewing tissue in the body. Lineage‐tracing studies have indicated that embryonic mammary epithelial cells (MECs) are multipotent *in vivo* 4, but their involvement in breast cancer is not yet clear. Tumours may develop from progenitor‐like cells at diverse stages of cellular differentiation 5, 6, 7, and embryonic mammary progenitor cells have potential links to breast cancer that remain to be explored 8.

We analysed embryonic mouse mammary gene signatures, and showed remarkable similarities between embryonic breast cells and breast cancer cells 9. We found that an embryonic mammary epithelial signature was activated in mouse *Brca1–*/^*–*^ tumours and human basal‐like breast cancers. A small network composed of embryonic genes with known roles in progenitor/stem cell regulation was found to be activated in some breast cancers. One network component, *SOX11*, is not detected in the normal mature postnatal breast, and is highly expressed in basal‐like and HER2+ breast cancers 9. SOX11 is known to promote tissue remodelling, progenitor cell expansion, and differentiation of a number of cell types 10, including neural progenitor cells 11. Induced expression of SOX11 in embryonic stem cells 12 and embryonic kidney cells 13 leads to induction of genes that regulate developmental processes, including organogenesis. *SOX11* expression within preinvasive breast lesions or invasive breast cancers may therefore indicate tissues containing cells that have distinct features that are more typically associated with prenatal mammary progenitor cells, and patterns of growth that are different from those characteristic of the mature postnatal breast 14, 15, 16.

Ductal carcinoma *in situ* (DCIS) presents a clinical problem, with risk of potential over‐ and undertreatment, and biomarkers are needed that can identify DCIS lesions that are likely to progress and require treatment with more aggressive therapies. *SOX11* has been found to be highly expressed in preinvasive lesions, including DCIS 17. Higher levels of *SOX11* have been detected in atypical ductal hyperplasia (ADH) that is associated with breast cancer than in ADH not associated with cancer 18. Therefore, SOX11 is an attractive candidate for mediating DCIS progression. Here, we analysed the effects of upregulating SOX11 expression in both mature breast cells and DCIS cells from the MCF10A progression series.

## Materials and methods

### Cell culture

Supplementary material, Table S1A provides details of cell lines and media. DCIS.com‐Luc cells were generated by transducing cells with lentiviral expression particles for firefly luciferase (LVP325; Amsbio, Abingdon, UK).

### Expression vectors

The *SOX11* coding sequence (GENEID: 6664) from clone HsCD00295480 19 in the pENTR223.1 plasmid (DNASU) 20 was subcloned into the pLenti6.3/V5‐DEST Gateway vector (ThermoFisher, Waltham, MA, USA).

### Flow cytometry analyses and fluorescence‐activated cell sorting (FACS)

After trypsinization, 5 × 10^4^ MCF10A or DCIS.com cells expressing SOX11 or control LacZ vector were resuspended in 100 μl of phosphate‐buffered saline (PBS) plus 10% fetal bovine serum (FBS), and incubated with combinations of antibodies, i.e. anti‐CD49f–PE (555736) (1:100), anti‐EpCAM–PerCPCy5.5 (347199) (1:20), anti‐CD24–FITC (555427) (1:20), anti‐CD24–PE–Cy7 (561646) (1:100) and anti‐CD44–APC (559942) (1:20) (BD Biosciences, Oxford, UK) for 30 min at room temperature. Cells were resuspended in 0.5 ml of PBS plus 10% FBS and 4′,6‐diamidino‐2‐phenylindole (DAPI), and filtered through 50‐μm filters. Unstained and single‐antibody‐stained cells were used for compensation. By use of a BD FACS LSRII flow cytometer, samples were analysed with BD FACS Diva software (BD Biosciences). Aldehyde dehydrogenase (ALDH) activity was measured with the Aldefluor assay (StemCell Technologies, Cambridge, UK); cells were also co‐stained with Aldefluor and CD49f–PE and EpCAM–PerCPCy5.5.

### Spheroid formation

Five thousand cells were plated in 96‐well ultra‐low‐attachment plates (Corning 7007, Corning, NY, USA), and spheroids formed after 4 days (supplementary material, Table S1A). Spheroid images were obtained starting on day 4 with a Celigo cytometer (Nexcelom, Manchester, UK).

### Clonogenic assays

MCF10A cells were plated at 250 per well in six‐well (Falcon F3046, Corning, NY, USA) plates. After 7 days, plates were stained with 0.2% crystal violet dissolved in 20% methanol in PBS. Clones were counted, and the percentage relative to number of cells plated was calculated.

For mammosphere assays, 5 × 10^3^ MCF10A cells/ml were plated in low‐attachment six‐well plates (Corning 3471) and incubated in medium (supplementary material, Table S1A) supplemented with 2% NeuroCult SM1 without vitamin A (StemCell Technologies) and 0.65% methylcellulose (R&D Systems, Abingdon, UK). After 14 days, wells were scanned with a Celigo cytometer (Nexcelom, Manchester, UK). Mammosphere‐forming efficiency was calculated by dividing the number of mammospheres by the number of cells plated per well.

### Immunofluorescence

Antibodies and staining protocols are detailed in supplementary material, Table S1B. Images were captured with a Leica Microsystems (Cambridge, UK) TCS‐SP2 confocal microscope.

### Western blotting

Western blotting was performed as previously described 9. Details of the antibodies used are provided in supplementary material, Table S1C.

### Transmigration, spheroid and invasion assays

DCIS.com cells were grown in serum‐free medium for 24 h and used in Cultrex 96‐well Collagen I Cell Invasion Assays (Amsbio, Abingdon, UK); cells (5 × 10^4^ per well) were plated in wells that had been coated with 1% or 0.1% collagen, or left uncoated. Transmigration was measured after 48 h.

Five thousand cells were plated per well in spheroid formation ECM medium within a 96‐well three‐dimensional (3D) spheroid BME cell invasion assay (3500‐096‐K; Cultrex). Matrix was added 3 days later, and culture medium was added 1 h after this (supplementary material, Table S1A). Spheres were measured every 2 days with a Celigo cytometer, and images were acquired to assess spheroid morphology.

3D spheroid invasion assays were performed. Spheroids from 5 × 10^3^ DCIS.com cells were embedded in collagen I (354249; Corning) at 2.2 mg/ml in serum‐free medium. Complete or serum‐free medium was added after 1 h. Images were acquired after 48 h with a Celigo cytometer. The total area of matrix invaded by cells was calculated with ImageJ after marking of the area manually.

### Proliferation and viability assays

Three thousand cells were plated in 96‐well plates (655098; Greiner Bio‐one, Stonehouse, UK) for 24 h. CellTiter‐Glo (Promega, Southampton, UK) was used according to the manufacturer's protocol. Luminescence was measured with a Victor X5 58 plate reader (Perkin‐Elmer, Seer Green, UK).

### Cleaved caspase‐3 (CC3) assays

Five thousand cells were plated in ultra‐low‐attachment 96‐well plates. Five days after spheroid formation for MCF10A cells, or 8 days after spheroid formation for DCIS.com cells, spheroids were incubated for 1 h with NucView‐488 Caspase‐3 substrate at a final concentration of 10 μm. Fluorescence was measured with the Celigo platform.

### Statistical analysis

Experiments were analysed with a two‐tailed Student's *t*‐test with a confidence interval of 95% when the number of groups equalled 2, or with a parametric anova and *post hoc* test when the number of groups was >2, unless otherwise specified.

### Animal experiments

SCID/beige mice, purchased from Charles River (Harlow, UK), were housed in individually ventilated cages on a 12‐h light/dark cycle, and received food and water *ad libitum*. All work was carried out under UK Home Office projects (70/7413 and 70/7712) and personal licenses (090/02921, I5F252069, and IFFDC436E) following receipt of local ethical approval from the Institute of Cancer Research Ethics Committee and in accordance with local and national guidelines. As biological replicates, four or five mice were used. Intraductal injections of 5 × 10^4^ cells were performed as previously described 21, with slight modifications, including the chemical removal of fur, and mice not being opened surgically. For mammary fat pad injections, 2.5 × 10^6^ cells were injected into mammary gland 4 of 10–12‐week‐old female mice. Engrafted mammary glands or tumours were harvested 6–12 weeks after intraductal injections and 6 weeks after mammary fat pad injections, and then fixed in formalin or snap‐frozen.

### RNA isolation

RNA was isolated from tumours or mammary gland 4 harbouring microinvasive lesions of each biological replicate (*n* = 4–5 for each time point) with TRIzol (Fisher Scientific, Loughborough, UK), and this was followed by a second extraction with an RNAeasyPlus Micro kit (74034; Qiagen, Manchester, UK) and DNase treatment. RNAClean and concentrator‐5 (Zymo Research, Irvine, CA, USA) were used. RNA concentration and purity were determined with a Qubit fluorometer (Invitrogen, Carlsbad, CA, USA) and a nanodrop spectrophotometer. RNA integrity number was measured with a bioanalyser and an Agilent RNA Pico kit (Agilent Technologies, Cheshire, UK).

### cDNA synthesis

One microgram of each RNA sample was reverse transcribed by use of a QuantiTect Reverse Transcription kit (Qiagen, Manchester, UK) in a final volume of 20 μl. cDNA was diluted eight‐fold for subsequent quantitative polymerase chain reaction (qPCR) analysis, as described previously 9, with the probes and methods listed in supplementary material, Table S2.

### Gene expression profiling

Sequence files were trimmed by the use of trim_galore (http://www.bioinformatics.babraham.ac.uk/projects/trim_galore/) with default settings. Trimmed data were separately mapped to the GRCh38 and GRCm38 genome assemblies by the use of hisat2 (v2.0.5) with options ‐‐sp 1000,1000 ‐‐ omixed‐‐no‐discordant, and were filtered to remove non‐primary alignments.

Species‐specific read sets were generated by removing any read that produced a valid alignment in both human and mouse from the results for both species. The remaining data were imported into SeqMonk (http://www.bioinformatics.babraham.ac.uk/projects/seqmonk/) with a filter of mapping quality (MAPQ) score ≥ 20. Reads were quantified over the transcript set from Ensembl v78 with annotated mis‐spliced, pseudogene and unannotated transcripts removed. Initial quantification was raw read counts from the opposing strand to the transcript, with all exons for each gene being collated into a single measure. This allowed gene‐level differential expression to be assessed by the use of DESeq2 (https://bioconductor.org/packages/release/bioc/html/DESeq2.html), with a cutoff of a false discovery rate of < 0.05. Subsequent visualization was performed by requantifying expression as log_2_ fragments per million reads of library. RNA sequencing data have been deposited in the European Nucleotide Archive with the accession number PRJEB19633.

### Immunohistochemistry (IHC)

IHC was performed on formalin‐fixed paraffin‐embedded DCIS samples from 22 oestrogen receptor (ER)+, 22 ER–, 17 HER2+ and six mixed cases collected through the ICICLE study with appropriate consent (MREC 08/H0502/4). Samples were stained with two SOX11 antibodies as described previously 9 (supplementary material, Table S3).

### Scoring guidelines

The expression status of SOX11 determined with the antibody Abcam ab170916 (Cambridge, UK) was assessed with a semiquantitative scoring system based on staining intensity and the proportion of positive cells expressed as a percentage. Staining intensity was divided into four grades (intensity scores): no staining (0), weak staining (1), moderate staining (2), and strong staining (3). The proportion of positive cells was divided into five grades (percentage scores): <10% (0), 10–25% (1), 26–50% (2), 51–75% (3), and 76–100% (4). SOX11 staining status was determined with the following formula: overall score = intensity score × percentage score. An overall score of ≤4 was defined as low expression; an overall score of >4 was defined as high expression.

### Survival analysis

The prognostic importance of *SOX11* mRNA expression was assessed by the use of survival data. Data obtained from the Gene Expression Omnibus, the European Genome‐phenome Archive and The Cancer Genome Atlas were examined by use of the Kaplan–Meier Plotter survival analysis tool (http://kmplot.com) and METABRIC 22, and statistical significance was determined with the Wald test and log‐rank test.

## Results

### Effect of SOX11 levels on postnatal mammary progenitor cell profiles

**Figure 1:**
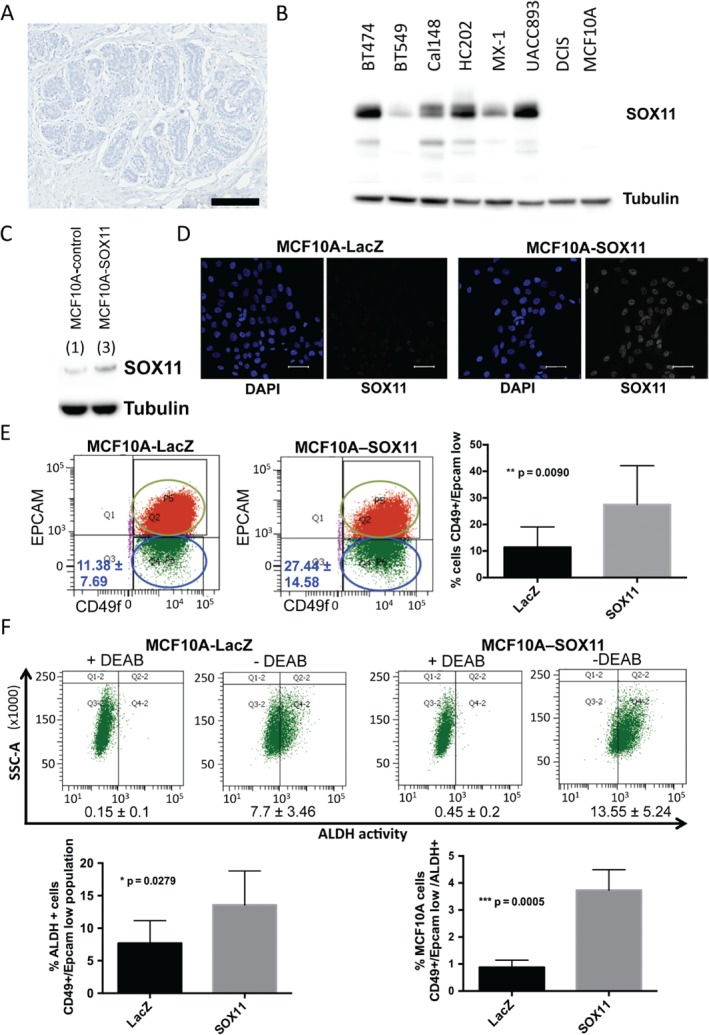
Expression of SOX11 in postnatal mammary epithelial cells alters progenitor cell populations. (A) SOX11 expression was not detected in normal mature breast tissue. Scale bar: 200 μm. (B) SOX11 was expressed in some basal‐like breast cancer and HER2+ cell lines, but not in MCF10A or DCIS.com cells. (C) Western blot of MCF10A‐LacZ control and MCF10A‐SOX11 cells. SOX11 levels (indicated by numerical values) were measured by densitometry, and normalized by dividing by the tubulin values. (D) Immunofluorescence staining of MCF10A‐LacZ control and MCF10A‐SOX11 cells with DAPI (blue in inset) and SOX11 (white). Scale bar: 50 μm. (E) Representative FACS analysis of EpCAM/CD49f‐sorted MCF10A‐control and MCF10A‐SOX11 cell populations. Experiments were performed five times. The average percentage of EpCAM–/CD49f + cells in each population is shown [median ± standard deviation (SD)]. Student's t‐test was performed. (F) ALDH activity levels in MCF10A‐control and MCF10A‐SOX11 cells were detected with the Aldefluor assay. Cells were stained and sorted with CD49f and EpCAM antibodies, and ALDH activity was measured with the Aldefluor kit. Representative ALDH activities after FACS analysis in EpCAM–/CD49f + MCF10A‐control and MCF10A‐SOX11 cell populations are shown. +DEAB plots display the negative control; cells incubated with diethylaminobenzaldehyde (DEAB), the specific inhibitor of ALDH, were used to establish the baseline fluorescence of these cells. Experiments were performed four times, and Student's t‐test was performed. The frequency of EpCAM–/CD49f + basal‐like ALDH+ cells (left graph) and EpCAM–/CD49f+/ALDH+ cells (right graph) in MCF10A‐SOX11 as compared with MCF10A‐LacZ control populations are shown. Error bars represent SD.

We confirmed that SOX11 is not expressed in normal mature breast tissue, as expected for an embryonic mammary marker (Figure 1A). We detected SOX11 expression in triple‐negative [ER–/progesterone receptor (PR)–/HER2–] and HER2+ breast cancer cell lines (Figure 1B). We did not detect significant expression of SOX11 in DCIS.com or MCF10A cells, which are ER– cell lines from a progression series that are often used as models to study DCIS and normal MEC growth, respectively (Figure 1B). To study the effects of expressing SOX11 in a normal MEC line, we stably transduced SOX11 into MCF10A cells (Figure 1C), and were able to detect nuclear SOX11 expression in MCF10A‐SOX11 cells (Figure 1D).

CD49f and EpCAM expression separates distinct subpopulations in cells isolated from human breast epithelium 23. Flow cytometry analysis with EpCAM and CD49f has also identified cell subpopulations in non‐tumourigenic basal cell lines 24. MCF10A cells show heterogeneity, and contain two separate subpopulations (Figure 1E): EpCAM+/CD49f + and EpCAM–/CD49f+. An increase in the EpCAM–/CD49f + basal‐like population was observed among MCF10A‐SOX11 cells (27.44 ± 14.58%) as compared with MCF10A‐control cells (11.38 ± 7.69%) (Figure 1E). ALDH activity was 1.76‐fold greater in EpCAM–/CD49f + basal‐like MCF10A‐SOX11 cells than in MCF10A‐control cells, suggesting an expanded population associated with stem cell properties (Figure IF; supplementary material, Figure S1). EpCAM–/CD49f+/ALDH+ cells were detected at 4.26‐fold greater frequency among MCF10‐SOX11 cells than among MCF10A‐control cells.

### Effect of SOX11 expression on postnatal MEC growth, morphogenesis, and clonogenicity

**Figure 2:**
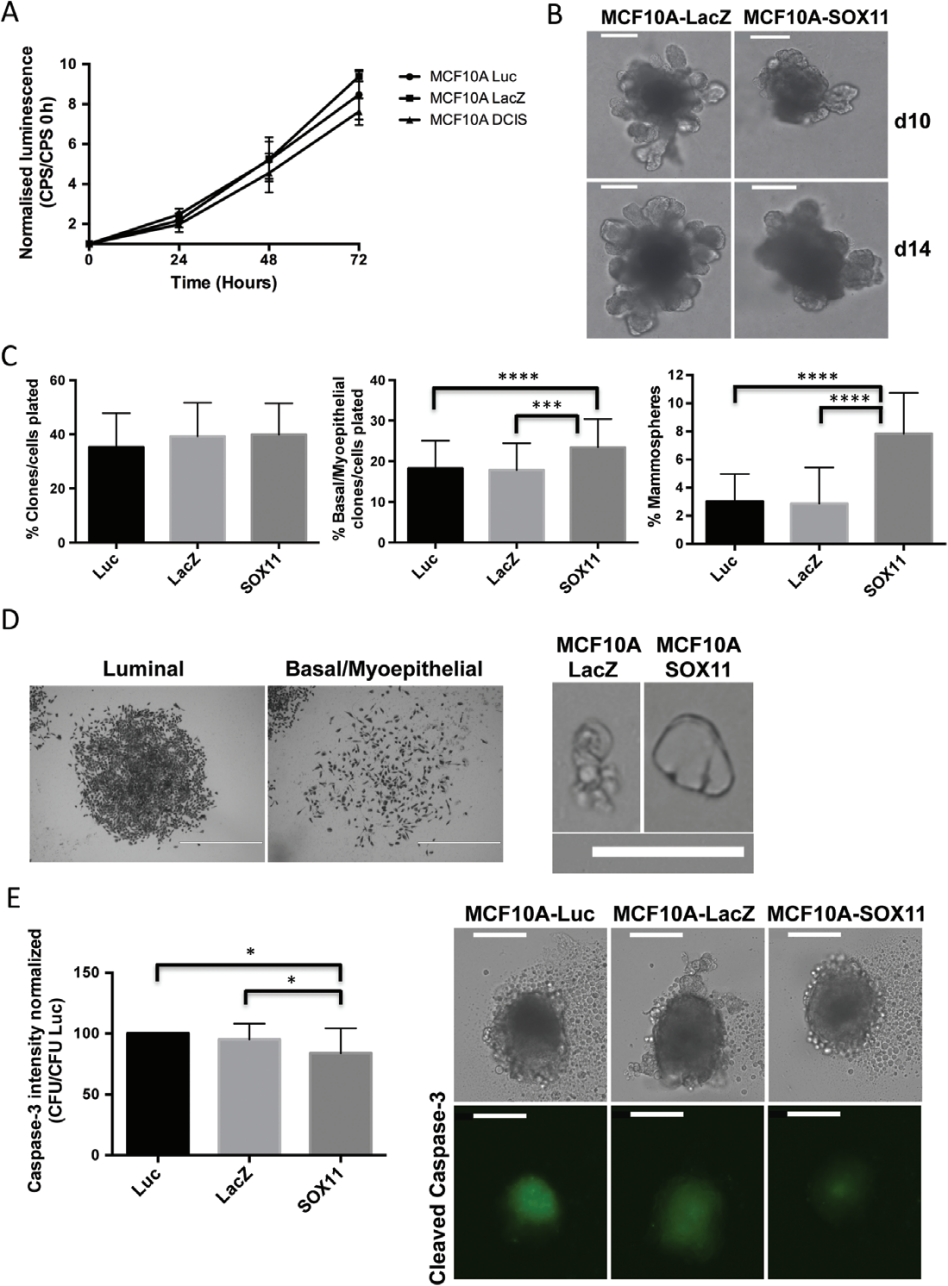
Effect of SOX11 expression on postnatal mammary epithelial cell growth, morphogenesis, and clonogenicity. (A) CellTiter‐Glo assay results for MCF10A‐LacZ‐control and MCF10A‐SOX11 cells. Experiments were performed three times (n = 18 in each sample), and anova and multiple comparisons were used for statistical analysis. The values obtained in each time point [counts per second (CPS)] were normalized by dividing by the value obtained at day 0 in each population. P < 0.05 for MCF10A‐SOX11 versus MCF10A‐LacZ; P < 0.0001 for MCF10A‐SOX11 versus MCF10A‐luc. (B) Representative images of MCF10A‐LacZ control and MCF10A‐SOX11 mammospheres that were grown from spheroids formed in low‐attachment plates, 10 and 14 days after addition of BME. Experiments were performed three times. Scale bar: 200 μm. (C) Quantification of clonogenicity and mammosphere‐initiating capacity. Left: percentage of MCF10A cell populations plated in two‐dimensional (2D) culture that form colonies. Centre: percentage of colonies with basal or myoepithelial morphology of the total cell number plated in 2D culture. Right: percentage of mammospheres formed from cells in 3D culture. All experiments were performed three times. ***P < 0.001, ***P < 0.0001. (D) Typical morphologies observed for colonies of MCF10A‐LacZ and MCF10A‐SOX11 cells in (C) (left) (scale bar: 1 mm) and for mammospheres derived from single MCF10A‐LacZ and MCF10A‐SOX11 cells embedded in methylcellulose (right). Single cells proliferated and formed cell clusters with a large central lumen in MCF10A‐SOX11 mammospheres. Scale bar: 200 μm. (E) Quantification of cleaved caspase‐3 levels (left) and representative images of MCF10A‐luc, MCF10A‐LacZ and MCF10A‐SOX11 spheroids 5 days after sphere formation. The experiment was performed three times; P < 0.05. Scale bar: 200 μm. All error bars represent standard deviation. CFU, Counts fluorescence units.

We observed a slight but significant reduction in cell growth in MCF10A‐SOX11 cells as compared with MCF10A‐LacZ cells (Figure 2A). Morphological differences were observed when MCF10A‐SOX11 mammospheres were compared with MCF10A‐LacZ control mammospheres grown under a variety of culture conditions; MCF10A‐SOX11 mammospheres appeared more compact and solid than controls (Figure 2B; supplementary material, Figure S2A, B). These observations are compatible with SOX11 altering the growth features of normal MECs, as we hypothesized on the basis of clinical data. MCF10A‐SOX11 cells are not more clonogenic, but form more clones with basal/myoepithelial morphology, than MCF10A‐control cells (Figure 2C, D). MCF10A‐SOX11 cells have greater mammosphere‐forming capacity and produce mammospheres with phenotypes that are distinguishable from those of MCF10A‐control cells (Figure 2C, D). CC3 levels were slightly reduced in MCF10A‐SOX11 mammospheres as compared with MCF10A‐control mammospheres (Figure 2E). No invasive growth was observed when MCF10A‐SOX11 spheroids were used in 3D invasion assays (supplementary material, Figure S2C). No morphological differences were observed between MCF10A‐SOX11 spheroids and MCF10A‐LacZ control spheroids grown without hydrogel (supplementary material, Figure S2D).

### Effect of SOX11 levels on DCIS

**Figure 3:**
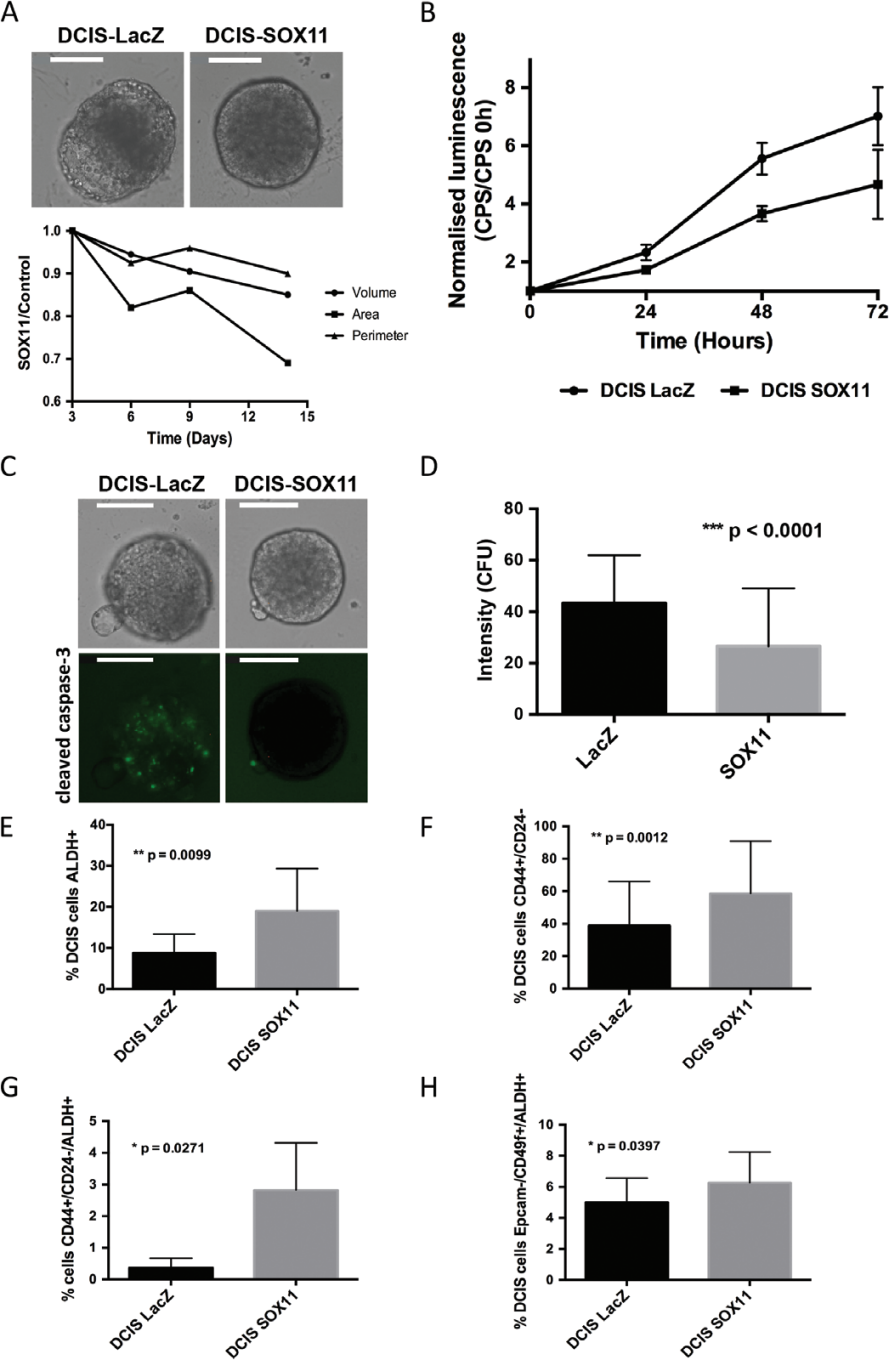
Effect of SOX11 expression on DCIS cells. (A) Spheroids from DCIS‐LacZ and DCIS‐SOX11 cells at day 14 grown in BME (top images): relative growth curves showing area (μm^2^), volume (μm^3^) and perimeter (μm) of DCIS‐SOX11 spheroids as compared with DCIS‐LacZ control spheroids. Scale bar: 200 μm. (B) CellTiter‐Glo assay results of DCIS‐LacZ control and DCIS‐SOX11 cells. Experiments were performed three times (n = 56 in each sample), and Student's t‐test was used for statistical analysis. The values obtained in each time point [counts per second (CPS)] were normalized by dividing by the value obtained at day 0 in each population. P < 0.0001 for the three time points. (C) Representative images of cleaved caspase‐3 activity in spheroids from DCIS‐LacZ control and DCIS‐SOX11 cells 8 days after spheres had formed. Scale bar: 200 μm. (D) Relative cleaved caspase‐3 activity detected in spheroids from DCIS‐SOX11 cells as compared with spheroids from DCIS‐LacZ cells at day 8 [Counts fluorescence units (CFU)]. (E) ALDH activity in DCIS‐control versus DCIS‐SOX11 populations. (F) Frequency of CD44+/CD24– cells in DCIS‐SOX11 as compared with DCIS‐control populations. (G) Frequency of CD44+/CD24–/ALDH+ cells in DCIS‐SOX11 as compared with DCIS‐control populations. (H) Frequency of EpCAM–/CD49f+/ALDH+ cells in DCIS‐SOX11 as compared with DCIS‐control populations. All error bars represent standard deviation.

We stably transduced SOX11 into DCIS.com cells to study the effects of expressing SOX11 in a cell line that is used to model DCIS formation and its progression to invasive disease (supplementary material, Figure S3). DCIS‐SOX11 spheroids appeared similar in morphology to DCIS‐control spheroids when they formed (day 0), but showed a slight reduction in volume after 14 days (Figure 3A). Cell growth was slightly reduced in DCIS‐SOX11 cells as compared with controls (Figure 3B). We assessed CC3 levels as a possible mechanism underlying the more compact and less necrotic phenotype observed in DCIS‐SOX11 spheroids. We detected lower levels of caspase‐3 activity in DCIS‐SOX11 spheroids than in DCIS‐control spheroids (Figure 3C, D). By FACS analysis, we detected over two‐fold higher ALDH activity in DCIS‐SOX11 cell populations than in controls (Figure 3E). The CD44+/CD24– population was increased 1.5‐fold in DCIS‐SOX11 cells (Figure 3F). A 7.7‐fold increase in the frequency of CD44+/CD24–/ALDH+ cells was detected among DCIS‐SOX11 cells as compared with control cell populations; no significant change was detected in the frequency of CD44+/CD24+/ALDH+ cells (Figure 3G; supplementary material, Figure S4). EpCAM–/CD49f+/ALDH+ cells were detected at a higher frequency among DCIS‐SOX11 cells than among controls (Figure 3H).

### SOX11 promotes invasive growth of DCIS cells in vitro

**Figure 4:**
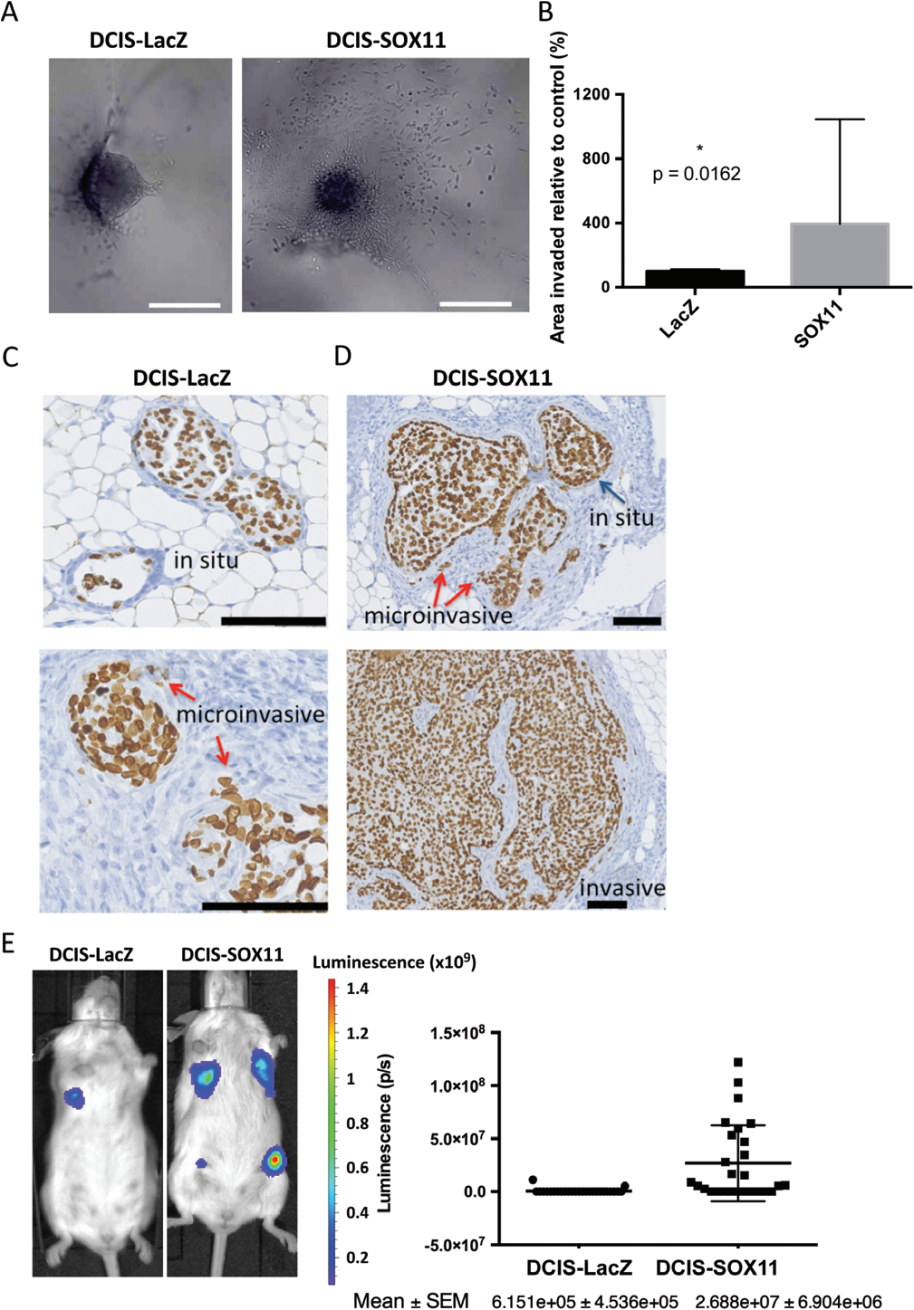
SOX11 increases the invasive activity of DCIS cells in vitro and promotes the growth and progression of DCIS cells in vivo. (A) Representative images of DCIS‐LacZ control and DCIS‐SOX11 spheroid invasion in collagen I at day 2. Scale bar: 500 μm. Error bars represent standard deviation. (B) Area of invasion of DCIS‐SOX11 as compared with DCIS‐LacZ control spheroids in collagen I at day 2. P = 0.0162. Experiments were performed three times. (C) Representative images of in situ and microinvasive lesions formed from DCIS‐LacZ control cells that appeared in mice injected intraductally 6–7 weeks after xenografting. Lamin A/C stain detects human cells. Scale bar: 100 μm. (D) Representative images of in situ lesions, microinvasive lesions and invasive lesions formed from DCIS‐SOX11 cells that appeared in mice injected intraductally 6–7 weeks after xenografting. Lamin A/C stain detects human cells. Scale bar: 100 μm. (E) Results from intraductal injections of DCIS‐LacZ control and DCIS‐SOX11 cells; representative images and quantification of in vivo bioluminescence 6–7 weeks after injection of DCIS‐LacZ control and DCIS‐SOX11 cells. Results are expressed in photons per second (p/s); P < 0.0001. Each dot represents the total photon count from each injected mammary gland. Error bars represent standard error of the mean (SEM).

Cell migration through collagen was significantly enhanced in DCIS‐SOX11 cells as compared with control cells in a Transwell assays (supplementary material, Figure S5A). The results from 3D invasion assays showed that SOX11 significantly increased invasion of DCIS‐spheroids through collagen (Figure 4A, B; supplementary material, Figure S5B). We measured levels of melanoma inhibitory activity (MIA), encoded by a gene in the PAM50 test, which are characteristically high in basal‐like breast cancer, and are decreased upon small interfering RNA‐mediated knockdown of *SOX11* in breast cancer cells, in a recent study 25. MIA is secreted by melanoma cells after their malignant transformation, and plays a key role in melanoma progression and invasion. We found that DCIS‐SOX11 cells expressed over four‐fold greater levels of MIA than DCIS‐LacZ control cells (supplementary material, Figure S6). Neither MCF10A‐LacZ nor MCF10A‐SOX11 cells expressed detectable levels of MIA. These results show that SOX11 expression can lead to profound phenotypic changes when expressed in mature ER–/PR–/HER2– breast cells, but the acquisition of an invasive phenotype is context‐dependent.

### SOX11 promotes growth and progression of DCIS cells in vivo

Using the mouse mammary intraductal (MIND) model developed by Behbod *et al*. 21, we injected DCIS‐control and DCIS‐SOX11 cells into the mammary ducts of female mice. We collected mammary glands 6–7 weeks later, and sectioned through a subset of them. Mammary glands from mice injected intraductally with DCIS‐control cells contained *in situ* lesions and some microinvasion (Figure 4C). Mammary glands from mice injected intraductally with DCIS‐SOX11 cells had extensive microinvasion and invasion (Figure 4D; supplementary material, Figure S7). We detected significantly more bioluminescence in mice injected intraductally with DCIS‐SOX11 cells 6–7 weeks after xenografting (Figure 4E). We also collected mammary glands from the same cohort of mice 12 weeks after xenografting; invasive tumours formed from both DCIS‐control and DCIS‐SOX11 cells. Significantly more bioluminescence was observed in DCIS‐SOX11 tumours, and these were larger than DCIS‐control tumours (supplementary material, Figure S7). RNA sequencing of lesions collected at the microinvasive stage (6–7 weeks after injection) showed deregulated RNA expression of extracellular matrix (ECM) components and cell shape regulators, and increased expression of secreted growth factors and peptides (Table 1). Invasive tumours collected 12 weeks after injection of DCIS cells into mammary ducts showed increased expression of genes encoding signal peptides, ECM components, and regulators of embryonic organ morphogenesis (Table 2). Downregulated genes in the lesions and tumours that formed after intraductal DCIS injections included those encoding glycoproteins, endopeptidase inhibitors, and regulators of neuron projection, apoptosis, and cell adhesion (supplementary material, Table S4).

**Table 1:** Potential downstream SOX11 targets identified by RNA sequencing of tumours formed after intraductal and fat pad injections of DCIS‐control and DCIS‐SOX11 cells; list of the top upregulated genes in DCIS‐SOX11 lesions from samples collected 6 weeks after intraductal mammary injection.

**Gene**	**Log**_**2**_ **FC** *
*FHAD1*	5.3392467
*UGT2B28*	4.79066443
*LRRC31*	4.1190376
*PIP*	3.9638702
*AGMO*	3.9168392
*TFAP2B*	3.7775288
*DUOX1*	3.6588801
*GSTM1*	3.6003848
*OLFM4*	3.2495492
*HHIPL2*	3.0788153
*SLC14A1*	3.0049578
*COX6B2*	2.9759872
*SIDT1*	2.4203497
*ALDH1A1*	2.1891004
*VAV3*	2.1827067
*HORMAD1*	2.0911084
*F5*	2.083366
*ELN*	2.029894
*SCGB1D2*	1.810388
*HSD17B2*	1.5279316
*FZD4*	1.5128815

* Log_2_FC indicates [log_2_(DCIS‐SOX11) – log_2_(DCIS‐control)].

**Table 2:** Potential downstream SOX11 targets identified by RNA sequencing of tumours formed after intraductal and fat pad injections of DCIS‐control and DCIS‐SOX11 cells; list of the top upregulated genes in DCIS‐SOX11 lesions from all intraductal samples (6 weeks and 12 weeks after mammary intraductal injections).

**Gene**	**Log**_**2**_ **FC** *
*FHAD1*	5.2158456
*DUOX1*	3.8396916
*GSTM1*	3.4844713
*FABP6*	3.4173422
*FILIP1*	2.9833927
*AGMO*	2.9238105
*QPRT*	2.9094748
*HLA‐DPA1*	2.8897848
*S100A7*	2.7633967
*FMOD*	2.6771894
*S100A8*	2.5575514
*KCNJ5*	2.5571022
*COX6B2*	2.5369272
*TFAP2B*	2.5153782
*XG*	2.385934
*PIP*	2.324072
*ROR2*	2.1787367
*SLC35F3*	2.0892406
*SLC4A8*	2.0528436
*FHL1*	2.0478764
*OLFM4*	2.0403762
*SHISA2*	2.028072
*TNFAIP6*	2.023
*NDRG4*	1.9837015
*TRIM6*	1.978967
*GAL3ST2*	1.8982594
*RINL*	1.8655803
*CLCA2*	1.8368406
*SYT12*	1.8143466
*VAV3*	1.7891986
*FBLN5*	1.7790818
*RMRP*	1.7490951
*APOC1*	1.7350571
*COL17A1*	1.7191944
*FLRT3*	1.6746507
*ARHGAP24*	1.639564
*ZNF503‐AS2*	1.6393551
*S100A9*	1.6338024
*HORMAD1*	1.6259812
*CFI*	1.5933018
*KCND1*	1.5714868
*TAF7L*	1.5505905
*RAD51AP2*	1.543372
*ELN*	1.5045118

* Log_2_FC indicates [log_2_(DCIS‐SOX11) – log_2_(DCIS‐control)].

We also injected DCIS‐control and DCIS‐SOX11 cells directly into the mammary fat pads of female mice. IVIS imaging showed that DCIS‐SOX11 cells had more bioluminescence and greater tumour volume 6 weeks after injection of cells (supplementary material, Figure S7). Invasive tumours that formed after mammary fat pad injections of DCIS‐SOX11 cells showed elevated expression of genes associated with organogenesis and developmental processes (supplementary material, Table S5), with similar gene signatures to those injected intraductally. A number of candidate effectors of SOX11 signalling in DCIS were identified, including *ALDH1A1* and *HORMAD1*, which have established links to breast cancer. ALDH1 is a marker of normal and malignant human mammary stem cells and a predictor of poor clinical outcome 26. *ALDH1A1* isoform expression is a cancer stem cell marker and predictor of progression and poor survival 27, 28. Elevated *HORMAD1* expression suppresses RAD51‐dependent homologous recombination and drives the use of alternative forms of DNA repair 29.

**Figure 5:**
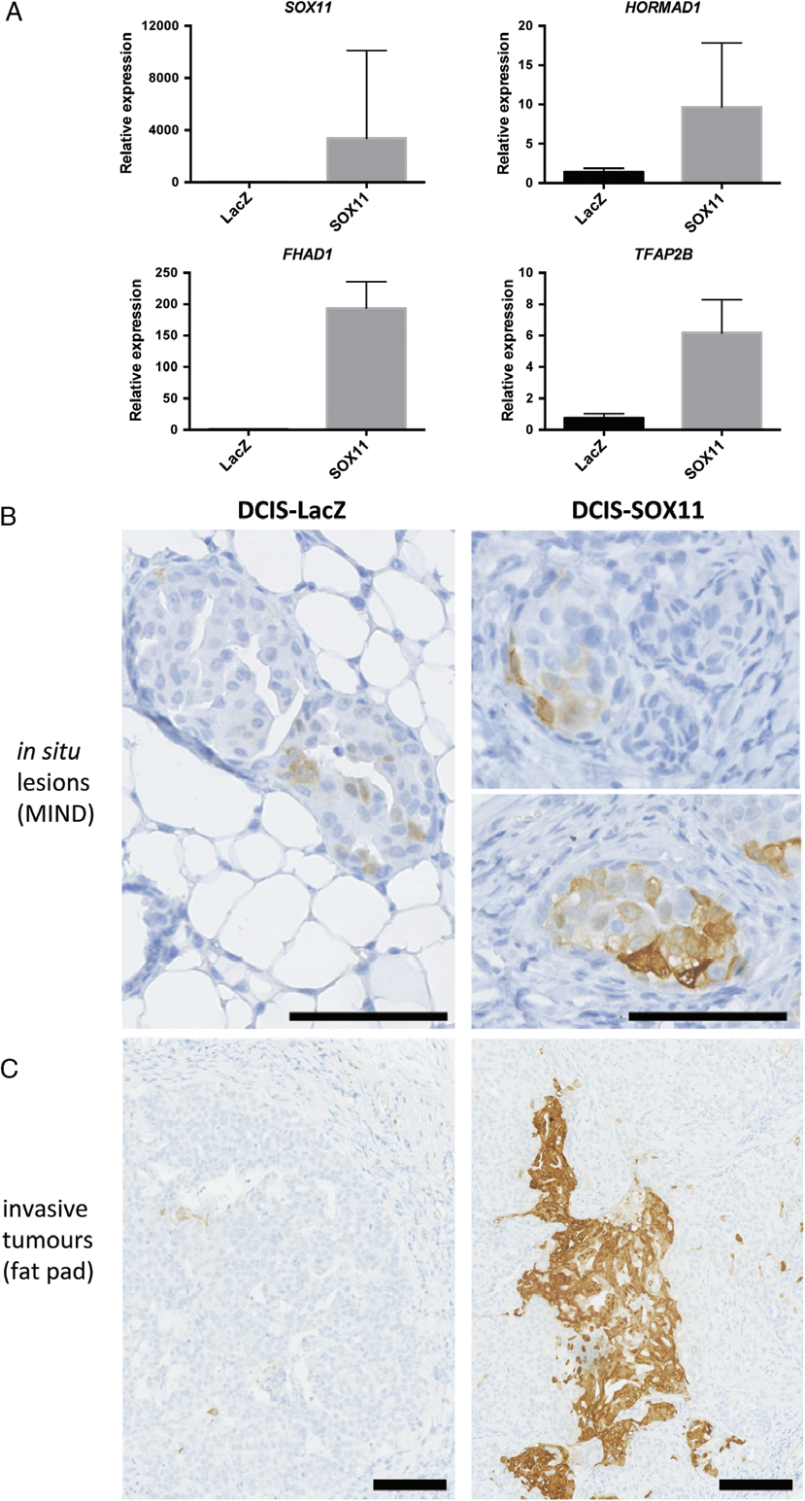
Downstream effectors of SOX11 signalling identified in a mouse model of DCIS that recapitulates human disease. (A) Quantitative polymerase chain reaction (qPCR) analysis of SOX11 and potential SOX11 downstream effectors, i.e. FHAD1, HORMAD1, and TFAP2B, in tumour samples; P < 0.0001. Results are expressed as fold change. Error bars represent standard deviation. (B) ALDH1A1 staining of DCIS lesions that form after mammary intraductal injections of DCIS‐LacZ control and DCIS‐SOX11 cells. Scale bar: 100 μm. (C) ALDH1A1 staining of tumours that formed after mammary fat pad injections of DCIS‐LacZ control and DCIS‐SOX11 cells. Scale bar: 100 μm.

Quantitative polymerase chain reaction (qPCR) analysis revealed higher levels of *SOX11*, *FHAD1*, *HORMAD1* and *TFAP2B* expression in DCIS‐SOX11 than in DCIS‐LacZ tumours (Figure 5A). We used IHC to stain ALDH1A1, and detected ALDH1A1+ cells in early lesions collected 6–7 weeks after injection of DCIS‐control cells and DCIS‐SOX11 cells (Figure 5B). In tumours formed after injection of DCIS‐control cells into the mammary fat pad, dispersed ALDH1A1+ cells were detected predominantly in the tumour interior (Figure 5C). We detected large clusters of ALDH1A1+ cells in the interior portion of the tumour, as well as at the tumour periphery, in DCIS‐SOX11 tumours (Figure 5C). ALDH1A1+ cells were detected in DCIS lesions from ER–/SOX11+ DCIS cases (supplementary material, Figure S8).

### SOX11 is expressed in breast cancers that progress to form metastases and in preinvasive breast lesions

**Figure 6:**
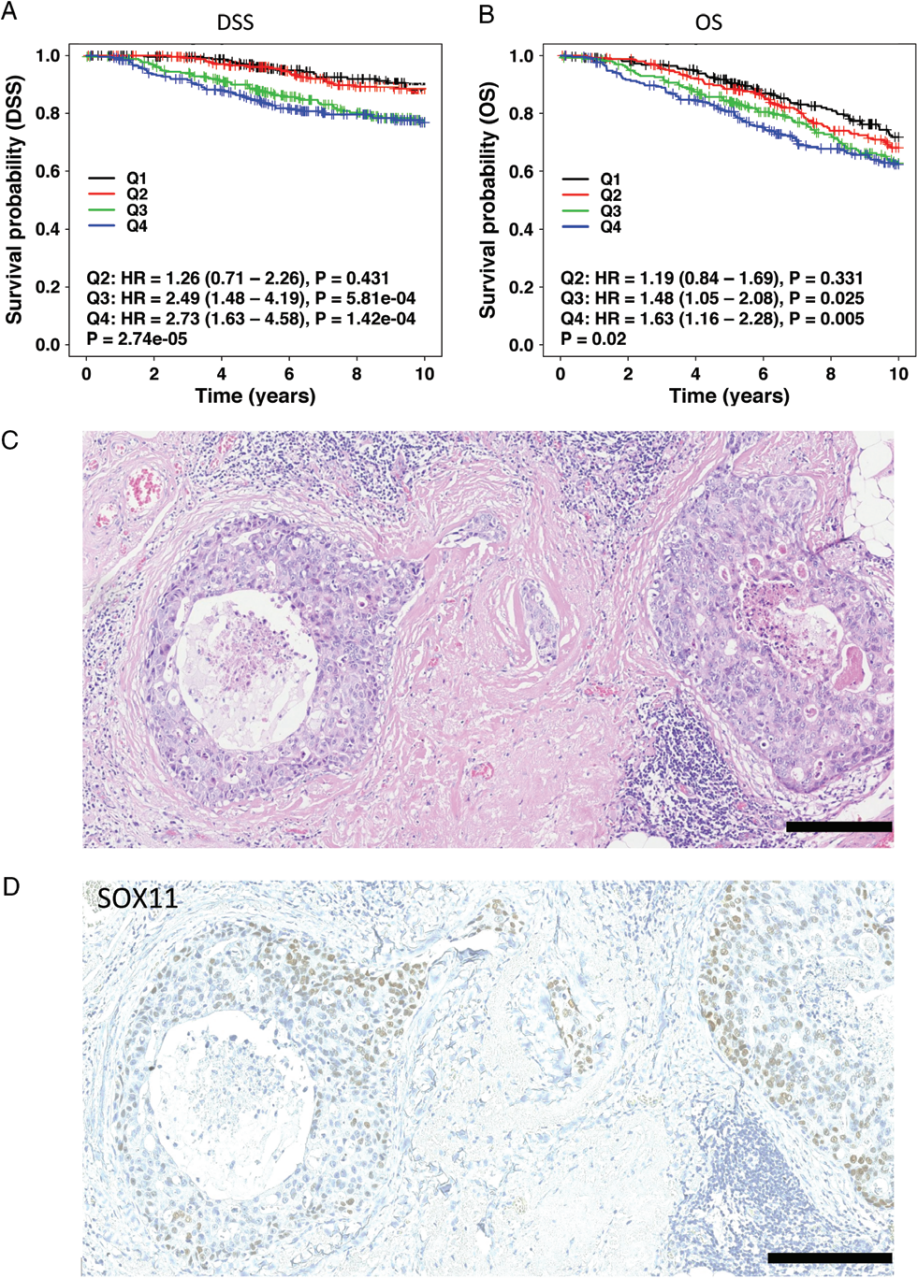
SOX11, an embryonic mammary epithelial marker, predicts poor clinical outcome in breast cancer patients and is expressed in preinvasive breast lesions. (A) Disease‐free survival (DSS) curves for 1032 breast cancer patients with lymph node‐negative disease and with low and high SOX11 expression from analysis of microarray data from the METABRIC dataset. Expression data were stratified into quartiles based on SOX11 expression, and the lowest expression quartile (Q1) was treated as the baseline for the subsequent pairwise comparisons with the remaining quartiles. The statistical significance of pairwise comparisons was assessed with the Wald test. (B) Overall survival (OS) curves for 1032 breast cancer patients with lymph node‐negative disease and with low and high SOX11 expression from analysis of microarray data from the METABRIC dataset. Expression data were stratified into quartiles based on SOX11 expression, and the lowest expression quartile (Q1) was treated as the baseline for the subsequent pairwise comparisons with the remaining quartiles. The statistical significance of pairwise comparisons was assessed with the Wald test. (C and D) Haematoxylin and eosin stain (C) and SOX11 expression (D) in DCIS lesions. Scale bar: 200 μm. HR, hazard ratio.

Higher levels of *SOX11* expression are associated with a worse outcome in patients with lymph node‐negative breast cancer, with an increased likelihood of the disease progressing to form distant metastases and decreased overall survival (Figure 6A, B; supplementary material, Figure S9) 30. High levels of nuclear SOX11 were detected by IHC in ER– DCIS lesions as compared with ER+ DCIS lesions (*P* = 0.0002, Fisher's exact test) in a small cohort of pure DCIS cases (13/22 ER–, six of 17 HER2+, one of 22 ER+, and DCIS and invasive components of four of six mixed cases) (Figure 6C, D; supplementary material, Figure S10).

## Discussion

We investigated the consequences of expressing SOX11, an embryonic mammary factor, in normal breast and DCIS cell lines originating from the MCF10A progression series. MCF10A and DCIS.com cells do not express significant levels of *SOX11* 31. Our findings show that engineering SOX11 expression in MCF10A cells significantly alters progenitor cell features and confers distinct traits on mature postnatal MECs. SOX11 expression results in an expanded basal‐like population. ALDH activity, a marker of human luminal progenitor cells 32, was increased significantly within the basal‐like population of MCF10A cells. These findings suggest that SOX11 expression in mature MECs increases the size of a population of cells with features of both basal and luminal lineages. A large percentage of prenatal mammary cells express markers associated with both the basal and luminal lineages in both mouse and human mammary epithelium 14, 33. An increased frequency of basal‐like clones and increased mammosphere‐forming capacity in MCF10‐SOX11 cells are consistent with the finding that ALDH+ cells from normal breast epithelium have stem cell properties 26. MCF10A‐SOX11 cells have a slight growth disadvantage as compared with controls, and do not show invasive properties. Expression of SOX11 in normal postnatal MECs alters progenitor cell features and morphogenesis without promoting invasion.

Expression profiling of the lesions and tumours that formed after intraductal injection of DCIS.com cells expressing SOX11 identified a large number of candidate downstream effectors, during both the microinvasive and invasive growth stages. Many have significant links to stem cell biology and embryonic developmental processes. ALDH1A1 plays a role in the proliferation and differentiation of mammary progenitor cells in the normal breast; clonogenicity is decreased upon *ALDH1A1* knockdown 34. TFAP2B is thought to stimulate cell proliferation and suppress terminal differentiation of specific cell types during embryonic development 35. Mutations in *TFAP2B* cause Char syndrome, a disorder characterized by defective heart, craniofacial and limb development 36. Cancer/testis genes are expressed by germ cells, and are quiescent in somatic cells, but activated in various cancers. *CT46*/*HORMAD1*, a meiotic gene, is a driver of homologous recombination deficiency in triple‐negative breast cancers 29.

Many genes found to be upregulated in DCIS‐SOX11 lesions encode ECM components or ECM modulators, including signal peptides and secreted growth factors. The ECM is highly modified in cancer, and can drive disease progression at the primary tumour site or its metastasis. SOX11 may contribute to the propagation of invasive phenotypic changes in DCIS.com cells by generating ECM cleavage products, including signal peptides with potential signalling functions and local release of growth factors. Together, our findings suggest that breast lesions expressing SOX11 have altered progenitor/stem cell populations and an increased propensity for tissue remodelling and invasion.

SOX11 is expressed in a variety of other cancers, including glioma, lung, mantle cell lymphoma (MCL), and ovarian and prostate cancer 37, 38. SOX11 is aberrantly expressed in most aggressive MCLs, and is considered to be a reliable biomarker in MCL pathology 39. *SOX11* is most highly expressed in basal‐like and HER2+ breast cancers, but further studies are needed to assess whether it will be a useful biomarker for clinical use 9, 25.

Our results show that the effect of SOX11 expression in mammary cells from the MCF10A series is context‐dependent. Invasive growth is dramatically increased in DCIS.com cells engineered to express SOX11, but not in MCF10A‐SOX11 cells. DCIS.com cells are clonally derived from H‐Ras‐transformed MCF10A cells, and it is plausible that, without expression of a driver of a malignant phenotype such as HRAS, SOX11 will not promote invasive growth. Three additional predicted cancer driver mutations (EPHA7, MAP3K12, and PCSK5) were identified that were acquired during the transformation of non‐malignant MCF10A cells to malignant DCIS.com cells that could also be implicated in the cell‐context dependency of SOX11 in promoting invasion 31.

A recent molecular study of DCIS and early‐stage invasive breast cancers detected high levels of *SOX11* expression in samples of pure DCIS and invasive tumours 40. High levels of *SOX11* expression were detected predominantly in basal‐like and HER2+ lesions. *SOX11* is coexpressed among a cluster of genes that identify a distinct DCIS subgroup with gene expression characteristics that are more similar to those of advanced tumours 41. Functional annotation of genes expressed within this DCIS subtype showed enrichment of genes associated with developmental processes and organ morphogenesis. Using *in vitro* and *in vivo* studies of DCIS.com cells, we showed that SOX11 promotes cell survival and invasion of DCIS cells, and increases the size of the ALDH+ population, including the CD44+/CD24–/ALDH+ subset. The CD44+/CD24–/ALDH+ phenotype is thought to increase tumourigenicity of breast cancer cells 26. Collectively, these findings support the notion that DCIS lesions that express *SOX11* possess properties that make them aggressive and cause them to progress to invasive breast cancer.

Of the ER– DCIS cases that we tested by IHC, 59% were SOX11+. High‐level *SOX11* expression is associated with poor overall survival in all breast cancer patients 9 and a poor outcome in patients with lymph node‐negative disease, a group that normally has a good predicted outcome. However, the datasets used for the survival analyses do not represent DCIS. In order to establish whether SOX11 overexpression in DCIS is associated with an increased risk of developing invasive disease, analysis of a large number of pure DCIS samples with long‐term follow‐up is required. The Sloane Project, a UK‐wide prospective audit of screen‐detected DCIS (http://www.sloaneproject.co.uk), and LORIS, a UK phase III trial comparing surgery with active monitoring for low‐risk DCIS, will be useful for evaluating the clinical value of SOX11 expression and its correlation with DCIS progressing to invasive breast cancer 42.

We have related SOX11 expression to increased invasive growth and progression of DCIS cells. We identified potential downstream effectors of SOX11 in DCIS, which adds new biological information that may contribute to a better understanding of the pathology and the identification of suitable treatment options for patients with breast lesions that express SOX11. SOX11 is a potential biomarker for ER– DCIS that may be at a higher risk of progression. Further investigations are needed to determine whether patients with DCIS lesions expressing SOX11 are more likely to develop invasive disease.

## Author contributions statement

The authors contributed in the following ways: EO, BAH: designed the experiments; EO, PB: performed *in vitro* experiments; NK, PB, BAH: performed *in vivo* experiments; DK, FD, PB, BAH: performed tumour staining and tumour scoring; SH: performed bioinformatics and survival analysis; VS, EJS: provided DCIS case samples and associated clinical data; BAH and EO: wrote the manuscript with input and approval from all authors.

**SUPPLEMENTARY MATERIAL ONLINE**

**Supplementary figure legends**

**Figure S1.** ALDH activity in bulk populations of MCF10A‐LacZ and MCF10A‐SOX11 cells

**Figure S2.** Examples of mammospheres formed from MCF10A‐LacZ and MCF10A‐SOX11 cells

**Figure S3.** Western blot of DCIS‐LacZ control and DCIS‐SOX11 cells

**Figure S4.** Frequency of CD44+/CD24+/ALDH+ cells in DCIS‐SOX11 compared to DCIS‐control populations

**Figure S5**. Results from invasion assays

**Figure S6.** Western blotting for MIA in DCIS‐LacZ control and DCIS‐SOX11 cells

**Figure S7.** Histology and bioluminescence data following intraductal xenografting of cells

**Figure S8**. A SOX11+ DCIS case immunostained for ALDH1A1

**Figure S9.** Relationships between *SOX11* expression and outcome

**Figure S10.** SOX11 and p63 expression in DCIS and invasive breast cancer

**Table S1**. Cell line details, media, antibodies, staining protocol

**Table S2**. Probes and protocol for RT‐qPCR

**Table S3**. Antibodies and conditions used for immunohistochemistry

**Table S4**. RNA‐sequencing data and results from functional annotation clustering of lesions and tumours from injection of DCIS‐LacZ and DCIS‐SOX11 cells into the mammary duct

**Table S5**. RNA‐sequencing data and functional annotation clustering of tumours from injection of DCIS‐LacZ and DCIS‐SOX11 cells into mammary fat pad

## Supporting information


**Supplementary figure legends**

**Figure S1.** ALDH activity in bulk populations of MCF10A‐LacZ and MCF10A‐SOX11 cells. Inset plots display the negative control; cells incubated with DEAB, the specific inhibitor of ALDH, were used to establish the baseline fluorescence of these cells.

**Figure S2.** Examples of mammospheres formed from MCF10A‐LacZ and MCF10A‐SOX11 cells. (A) Mammospheres that form when spheroids are embedded in BME after spheroid formation. Day 1, 3 and 5 after BME mixed with SFM was added to spheroids with complete media on top. (B) Mammospheres that form when spheroids are embedded in BME after spheroid formation. Day 0 and 18 after BME mixed with SFM was added with SFM on top. (C) Spheroids embedded in 2.2mg/ml of Collagen I with complete media added on top at Day 0 and Day 3. (D) Spheroids formed in low attachment plates in normal media at days 4, 7, 10 and 14 (no hydrogel). Day 0 = 4 days after plating cells to form spheroids on low attachment plates in A‐D.

**Figure S3.** Western blot of DCIS‐LacZ control and DCIS‐SOX11 cells. The levels of SOX11 were measured by densitometry and normalised dividing by the tubulin values.

**Figure S4.** Frequency of CD44+/CD24+/ALDH+ cells in DCIS‐SOX11 compared to DCIS‐control populations.

**Figure S5**. Results from invasion assays. (A) Results from Transwell invasion assays of DCIS‐LacZ control and DCIS‐SOX11 cells through 0.1% Collagen. (units are counts per second (cps)), p=0.0014**.** Experiment was performed three times.

**Figure S6.** Western blotting for MIA in DCIS‐LacZ control and DCIS‐SOX11 cells. The levels of MIA were measured by densitometry and normalised dividing by the tubulin values.

**Figure S7.** Histology and bioluminescence data following intraductal xenografting of cells. (A) Mammary glands were collected six wk after intraductal injection. Samples from each cohort (DCIS‐LacZ and DCIS‐SOX11) were fixed in formalin and embedded in paraffin wax. One mammary gland from the first three mice that had been embedded from each cohort were sectioned and scored for presence of in situ, microinvasive and invasive lesions. (B) Tumours volumes from four mammary glands from each cohort (DCIS‐LacZ and DCIS‐SOX11) collected twelve wk after intraductal injections. p=0.0286. Mann‐Whitney test was used. (C) Results from mammary fat pad injections of DCIS‐LacZ control and DCIS‐SOX11 cells. Representative images and quantification of in vivo bioluminescence six wk after injection of DCIS‐LacZ control and DCIS‐SOX11 cells. Results expressed in photons per second (p/s); p=0.0034. (D) Tumours volumes from mammary glands from each cohort (DCIS‐LacZ and DCIS‐SOX11) collected six wk after mammary fat pad injections. p=0.1111. Mann‐Whitney test was used.

**Figure S8**. A SOX11+ DCIS case immunostained for ALDH1A1. Scale bar: 200 μm

**Figure S9.** Relationships between SOX11 expression and outcome. (A) Distant metastasis‐free survival (DMFS) curves for breast cancer patients with lymph node negative disease with low and high SOX11 expression from analysis of microarray data of 988 patients using Kaplan‐Meier Plotter survival analysis tool (http://kmplot.com). Expression data was dichotomised compared to the highest quartile expression level. (B) Overall survival (OS) curves for breast cancer patients with lymph node negative disease with low and high SOX11 expression from analysis of microarray data of 594 patients using the Kaplan‐Meier Plotter survival analysis tool (http://kmplot.com). Expression data was dichotomised compared to the highest quartile expression level.

**Figure S10.** SOX11 and p63 expression in DCIS and invasive breast cancer. (A) H&E stain, SOX11 and p63 expression in DCIS lesions from a mixed ER‐, HER2+ case with high grade DCIS. scale bar: 100μm. (B) H&E stain, SOX11 and p63 expression in invasive breast cancer from a mixed ER‐, HER2+ case with high grade DCIS (DCIS shown in A). scale bar: 100 μm

**Table S1.** Antibodies used in Western blots

**Table S2.** Probes and protocol for RT‐qPCR

**Table S3.** Antibodies and conditions used for Immunohistochemistry

**Table S4.** Upregulated genes in lesions and tumours from DCIS‐SOX11 cells compared to DCIS‐lacZ cells injected into the mammary duct.

**Table S5.** Functional annotation clustering of upregulated genes in tumours from DCIS‐SOX11 cells compared to DCIS‐lacZ cells injected into mammary fat pad.
