# Supplementary material for: SOX11 promotes invasive growth and ductal carcinoma in situ progression
Source: J Pathol. 2017 Aug 22;243(2):193–207. doi: 10.1002/path.4939 (PMC5637904; doi:10.1002/path.4939)
Supplement: Supplementary file 1 — Supplementary figure legends [file PATH-243-193-s001.doc]

**Supplementary figure legends**

**Figure S1.** ALDH activity in bulk populations of MCF10A-LacZ and MCF10A-SOX11 cells. Inset plots display the negative control; cells incubated with DEAB, the specific inhibitor of ALDH, were used to establish the baseline fluorescence of these cells.

**Figure S2.** Examples of mammospheres formed from MCF10A-LacZ and MCF10A-SOX11 cells. (A) Mammospheres that form when spheroids are embedded in BME after spheroid formation. Day 1, 3 and 5 after BME mixed with SFM was added to spheroids with complete media on top. (B) Mammospheres that form when spheroids are embedded in BME after spheroid formation. Day 0 and 18 after BME mixed with SFM was added with SFM on top. (C) Spheroids embedded in 2.2mg/ml of Collagen I with complete media added on top at Day 0 and Day 3. (D) Spheroids formed in low attachment plates in normal media at days 4, 7, 10 and 14 (no hydrogel). Day 0 = 4 days after plating cells to form spheroids on low attachment plates in A-D.

**Figure S3.** Western blot of DCIS-LacZ control and DCIS-SOX11 cells. The levels of SOX11 were measured by densitometry and normalised dividing by the tubulin values.

**Figure S4.** Frequency of CD44+/CD24+/ALDH+ cells in DCIS-SOX11 compared to DCIS-control populations.

**Figure S5**. Results from invasion assays. (A) Results from Transwell invasion assays of DCIS-LacZ control and DCIS-SOX11 cells through 0.1% Collagen. (units are counts per second (cps)), p=0.0014**.** Experiment was performed three times.

(B) Representative images of invasion from spheroids (red areas circled in yellow) grown in serum-free (SF) or serum-containing (+serum) media 48 h after addition of Collagen I and invasion media. ‘Area invaded’ at the end point includes area at T=0.

**Figure S6.** Western blotting for MIA in DCIS-LacZ control and DCIS-SOX11 cells. The levels of MIA were measured by densitometry and normalised dividing by the tubulin values.

**Figure S7.** Histology and bioluminescence data following intraductal xenografting of cells. (A) Mammary glands were collected six wk after intraductal injection. Samples from each cohort (DCIS-LacZ and DCIS-SOX11) were fixed in formalin and embedded in paraffin wax. One mammary gland from the first three mice that had been embedded from each cohort were sectioned and scored for presence of *in situ*, microinvasive and invasive lesions. (B) Tumours volumes from four mammary glands from each cohort (DCIS-LacZ and DCIS-SOX11) collected twelve wk after intraductal injections. p=0.0286. Mann-Whitney test was used. (C) Results from mammary fat pad injections of DCIS-LacZ control and DCIS-SOX11 cells. Representative images and quantification of *in vivo* bioluminescence six wk after injection of DCIS-LacZ control and DCIS-SOX11 cells. Results expressed in photons per second (p/s); p=0.0034. (D) Tumours volumes from mammary glands from each cohort (DCIS-LacZ and DCIS-SOX11) collected six wk after mammary fat pad injections. p=0.1111. Mann-Whitney test was used.

**Figure S8**. A SOX11+ DCIS case immunostained for ALDH1A1. Scale bar: 200 µm

**Figure S9.** Relationships between *SOX11* expression and outcome. (A) Distant metastasis-free survival (DMFS) curves for breast cancer patients with lymph node negative disease with low and high *SOX11* expression from analysis of microarray data of 988 patients using Kaplan-Meier Plotter survival analysis tool (http://kmplot.com). Expression data was dichotomised compared to the highest quartile expression level. (B) Overall survival (OS) curves for breast cancer patients with lymph node negative disease with low and high *SOX11* expression from analysis of microarray data of 594 patients using the Kaplan-Meier Plotter survival analysis tool (http://kmplot.com). Expression data was dichotomised compared to the highest quartile expression level.

**Figure S10.** SOX11 and p63 expression in DCIS and invasive breast cancer. (A) H&E stain, SOX11 and p63 expression in DCIS lesions from a mixed ER-, HER2+ case with high grade DCIS. scale bar: 100µm. (B) H&E stain, SOX11 and p63 expression in invasive breast cancer from a mixed ER-, HER2+ case with high grade DCIS (DCIS shown in A). scale bar: 100 µm
